# Pharmacodynamic effect of a new γ‐secretase modulator, RG6289, on CSF amyloid‐β peptides in a randomized Phase I study

**DOI:** 10.1002/alz.095213

**Published:** 2025-01-09

**Authors:** Thomas Mueggler, Agnes Portron, Agnès Poirier, Annamarie Vogt, Tianxu Yang, Adnan Mohamed Abdi, Gwendlyn Kollmorgen, Norbert Wild, Cory Simmons, Kalbinder Mahil, Lothar Lindemann, Karlheinz Baumann, Taner Varda, Rosanna Tortelli, Irene Gerlach, Stefan Sturm

**Affiliations:** ^1^ Neuroscience and Rare Diseases, Roche Pharma Early Research and Development, F. Hoffmann‐La Roche Ltd, Basel Switzerland; ^2^ Pharmaceutical Sciences, Roche Pharma Research and Early Development, F. Hoffmann‐La Roche Ltd, Basel Switzerland; ^3^ Neuroscience and Rare Diseases, Roche Pharma Early Research and Development, F. Hoffmann‐La Roche Ltd, Basel, Switzerland, Basel Switzerland; ^4^ Product Development Safety Risk Management, F. Hoffmann‐La Roche Ltd, Roche China Holding, Beijing China; ^5^ Roche Diagnostics GmbH, Penzberg Germany; ^6^ Product Development Data Sciences, F. Hoffmann‐La Roche Ltd, Mississauga, ON Canada; ^7^ Roche Innovation Center Welwyn, Roche Pharma Research and Early Development, Roche Products Limited, Welwyn United Kingdom; ^8^ Product Development Safety Risk Management, F. Hoffmann‐La Roche Ltd, Basel Switzerland

## Abstract

**Background:**

γ‐Secretase modulators (GSMs) represent a promising therapy for Alzheimer’s disease (AD). GSMs selectively reduce amyloidogenic long Aβ peptides (e.g.: Aβ42) while simultaneously increasing shorter, non‐amyloidogenic, Aβ species (Aβ38 and Aβ37). Here we report the results of pharmacodynamic (PD) effects of RG6289, a novel, potent and selective, orally bioavailable GSM, in a Entry‐in‐Human (EiH) Phase I study including data from serial cerebrospinal fluid (CSF) sampling.

**Method:**

In a Phase I study in healthy volunteers, the PD effects of RG6289 were investigated following single and multiple ascending oral doses of RG6289 or placebo. Plasma and CSF samples were collected over 36 hours after single dose administration in a dedicated serial CSF sampling part of the study. Samples were analyzed using the Elecsys® plasma and CSF assays to assess effects of RG6289 on Aβ metabolism (Aβ42, Aβ40, Aβ38, Aβ37)

**Result:**

For serial CSF sampling, 12 healthy volunteers (46‐70 years) were randomized to receive a single dose of RG6289 (n = 9) or placebo (n = 3). Data from serial CSF sampling were highly variable and concentrations of all Aβ monomers increased over the sampling period after administration of placebo. A dose‐dependent increase in Aβ37/Aβ40 and Aβ38/Aβ42 ratios was observed up to 36 hours post dose. At the highest dose, an approximate 500% and 150% increase from baseline in the ratio of Aβ37/Aβ40 and Aβ38/Aβ42, respectively, was measured. Multiple ascending oral administration of RG6289 for 2 weeks resulted in an increase in Aβ37, Aβ37/Aβ40, Aβ38 and Aβ38/Aβ42 up to approximately 350%, 1100%, 40% and 400%, respectively, whilst there was a decrease in Aβ40 and Aβ42 of up to approximately 60% and 70%, respectively.

**Conclusion:**

In humans, RG6289 produced a dose‐dependent shift in the production of longer (Aβ40 and Aβ42) and shorter (Aβ37 and Aβ38) Aβ monomers in CSF. The results from this study support the further clinical development of RG6289 for the treatment of AD with the goal to reduce production of Aβ42, increase the levels of smaller Aβ species and slow‐down or halt Aβ aggregation and accumulation as an approach to altering AD progression.

